# Sex Differences in the Risk of Alzheimer’s Disease and Associated Risk Factors Among Aging Hispanics: A Cross‐country Study

**DOI:** 10.1002/alz.092718

**Published:** 2025-01-09

**Authors:** Yuexuan Xu, Dolly Reyes‐Dumeyer, Cory Weinfeld, Angel Piriz, Danurys Sanchez, Belisa Soriano, Yahaira Franco, Zoraida Dominguez Coronado, Patricia Recio, Diones Rivera Mejia, Martin Medrano, Rafael A. Lantigua, Badri N. Vardarajan, Richard Mayeux

**Affiliations:** ^1^ Columbia University, New York, NY USA; ^2^ G. H. Sergievsky Center, Vagelos College of Physicians and Surgeons, Columbia University, New York, NY USA; ^3^ The Taub Institute for Research on Alzheimer’s Disease and the Aging Brain, Vagelos College of Physicians & Surgeons, Columbia University, New York, NY USA; ^4^ Columbia University Medical Center, New York, NY USA; ^5^ Universidad Pedro Henríquez Urena, Santo Domingo Dominican Republic; ^6^ Clinica Corominas, Santiago Dominican Republic; ^7^ Clinica Gregorio Hernandez, Puerta Plata Dominican Republic; ^8^ CEDIMAT, Santo Domingo Dominican Republic; ^9^ CEDIMAT, Santo Domingo, Santo Domingo Dominican Republic; ^10^ School of Medicine, Pontificia Universidad Catolica Madre y Maestra, Santiago Dominican Republic; ^11^ Columbia University Irving Medical Center, New York, NY USA; ^12^ The Gertrude H. Sergievsky Center, College of Physicians and Surgeons, Columbia University, New York, NY USA; ^13^ Gertrude H. Sergievsky Center, Vagelos College of Physicians & Surgeons, Columbia University, New York, NY USA; ^14^ Department of Neurology, Columbia University Medical Center, New York, NY USA

## Abstract

**Background:**

Hispanics, one of the fastest‐growing populations in the United States, are disproportionately affected by Alzheimer’s disease (AD). Understanding the variations in AD risk associated with sex and the impact of relocation from their home country is crucial in designing interventions to address health disparities. This study explores the differential impact of sex and geographic relocation on dementia risk and its associated factors among Hispanics.

**Method:**

We utilized data from two observational samples, encompassing 4,960 individuals from the Estudio Familiar de la Influencia Genética en Alzheimer (EFIGA), primarily based in the Dominican Republic, and 2,614 individuals enrolled in the Washington Heights‐Hamilton Heights‐Inwood Community Aging Project (WHICAP), based in the US. In both, we evaluated AD‐related risk factors and biomarker concentrations by sex and disease status, using two‐sample t‐tests and chi‐squared tests. We assessed AD risk across the full sample and conducted sex‐stratified logistic regression analyses, along with linear regression to compare biomarker concentrations and disease onset age among sexes.

**Results:**

In the EFIGA study, the risk of AD was found to be lower among women (Odds Ratio [OR] = 0.86, 95% Confidence Interval [CI]: 0.75‐0.98), whereas in WHICAP, no significant sex‐based differences were observed. Concerning the age of onset, women in EFIGA exhibited an earlier onset (β = ‐1.42, *p* < 0.001), contrasting with a later onset in WHICAP (β = 1.02, *p* = 0.007). In the sex‐stratified analyses, both men (OR = 1.66, 95% CI: 1.32‐2.09) and women (OR = 1.64, 95% CI: 1.40‐1.91) in EFIGA showed distinct associations of AD with *APOE* ε4, a pattern only seen among women in WHICAP (OR = 1.34, 95% CI: 1.06‐1.68). With respect to biomarker concentrations, sex‐related differences were noted in Ab42/40 ratio, total tau, phosphorylated tau, glial fibrillary acidic protein, and neurofilament light, although the specific patterns varied between WHICAP and EFIGA.

**Conclusion:**

Sex‐related differences in AD risk and associated factors among Hispanics, were evident in both EFIGA and WHICAP studies, and are potentially further influenced by geographic relocation and migration patterns.

